# The effects of greenness exposure on hypertension incidence among Chinese oldest-old: a prospective cohort study

**DOI:** 10.1186/s12940-022-00876-6

**Published:** 2022-07-11

**Authors:** Zhou Wensu, Wang Wenjuan, Zhou Fenfen, Chen Wen, Ling Li

**Affiliations:** Department of Medical Statistics, School of Public Health, Sun Yat-sen University, Guangzhou, China

**Keywords:** Greenness, China, Hypertension, Oldest-old population, Cohort study

## Abstract

**Background:**

Although the oldest-old (those aged over 80 years) are vulnerable to environmental factors and have the highest prevalence of hypertension, studies focusing on greenness exposure and the development of hypertension among them are insufficient. The aim of this study was to explore the association between residential greenness and hypertension in the oldest-old population.

**Methods:**

This cohort study included data from the Chinese Longitudinal Healthy Longevity Survey (CLHLS). The oldest-old were free of hypertension at baseline (2008), and hypertension events were assessed by follow-up surveys in 2011, 2014, and 2018. The one-year averages of the normalized difference vegetation index (NDVI) and enhanced vegetation index (EVI) at 500-m buffer before the interview year of incident hypertension or last censoring interview were collected at the level of 652 residential units (district or county). The linear or nonlinear association between greenness and hypertension incidence was analyzed using the Cox proportional hazards model with penalized splines. The linear links between greenness and hypertension incidence were determined using the Cox proportional hazards model included a random effect term.

**Results:**

Among 5253 participants, the incidence rate of hypertension was 7.25 (95% confidence interval [CI]: 6.83–7.67) per 100 person-years. We found a nonlinear association between greenness exposure and hypertension risk, and the exposure-response curve showed that 1 change point existed. We examined the linear effect of greenness on hypertension by categorizing the NDVI/EVI into low and high-level exposure areas according to the change point. We found more notable protective effects of each 0.1-unit increase in greenness on hypertension incidence for participants living in the high-level greenness areas (hazard ratio (HR) = 0.60; 95% CI: 0.53–0.70 for NDVI; HR = 0.46; 95% CI: 0.37–0.57 for EVI). In contrast, no significant influence of greenness exposure on hypertension risk was found for participants living in the low-level greenness areas (HR = 0.77; 95% CI: 0.38–1.55 for NDVI; HR = 0.73; 95% CI: 0.33–1.63 for EVI).

**Conclusions:**

Greenness exposure is nonlinearly associated with hypertension risk among the oldest-old, presenting its relationship in an inverse “U-shaped” curve. Greenness is a protective factor that decreases the risk of hypertension.

**Supplementary Information:**

The online version contains supplementary material available at 10.1186/s12940-022-00876-6.

## Background

Greenness usually comprises vegetation and is closely associated with natural elements [1]; it is a widely used environmental indicator in the medical and health sciences field. An increasing number of studies have confirmed that greenness is beneficial for cardiovascular health [2, 3]. Greenness can offset the effects of environmental hazards and reduce damage to the cardio-cerebral vascular system caused by air pollutants, heat, and noise; greenness exposure can relieve mental stress as well as help reduce the proportion of overweight and obese individuals [4].

Currently, the rapidly increasing aging population, including the expanding oldest-old population size (i.e., those aged ≥80 years), has resulted in a large cardiovascular disease (CVD) burden [5, 6]. Hypertension, which is well known as an important contributor to CVDs, is not only the leading preventable risk factor for CVDs but also has the highest prevalence among the oldest-old [7, 8]. Efforts to identify factors that influence hypertension incidence are critical for reducing the disease burden of CVDs and promoting healthy aging.

Previous studies have revealed a negative association between greenness and hypertension risk [3, 9–11]; however, several questions remain unanswered. First, numerous studies have revealed the association between greenness and hypertension in older adults, but the results were inconsistent. For instance, two surveys showed no significant association between greenness exposure and blood pressure (BP) in older adults [12, 13]. Second, published studies have suggested that the relationship between greenness and BP showed a modification effect that varied by the age of older adults. Some studies proposed that this protective effect was stronger in the younger age group, but others did not. However, few studies have indicated this association in older adults aged ≥ 80 years [10, 11, 14], although the prevalence of hypertension is quite high in the oldest-old, and they are more vulnerable to environmental risk factors owing to organ function decline [15]. In addition, previous cross-sectional study designs dominate the literature [3, 16, 17]. There is little data regarding the prospective effects of greenness exposure on the incidence of hypertension, implying a restricted ability when predicting outcomes. Furthermore, many published studies have been limited to local areas with mostly small sample sizes that may not be properly representative [18, 19].

Importantly, the recommendations in the guidelines for the prevention of geriatric hypertension are updated every few years, relying on newer evidence for the influence of related factors on hypertension. However, far less attention has been paid to environmental factors in the practice guidelines for older adults. Environmental factors are omnipresent and have clinically meaningful effects on hypertension [20]. With progressively aging societies and the prevalence of geriatric hypertension continues to increase, a deeper understanding of the association between greenness and hypertension in specific older adults is needed.

Therefore, the objective of this study was to examine the association between greenness exposure and hypertension incidence based on a prospective cohort study of older adults aged ≥80 years in China.

## Methods

### Study design and population

The data were derived from the public open database Chinese Longitudinal Healthy Longevity Survey (CLHLS), which was a national investigation that recruited older adults aged ≥ 65 years from 23 of 31 provinces, autonomous regions, and municipalities in mainland China. Since the first investigation (1998), the CLHLS had conducted eight investigations by the 2018 wave. A detailed introduction to CLHLS has been published elsewhere [21].

**Fig. 1:**
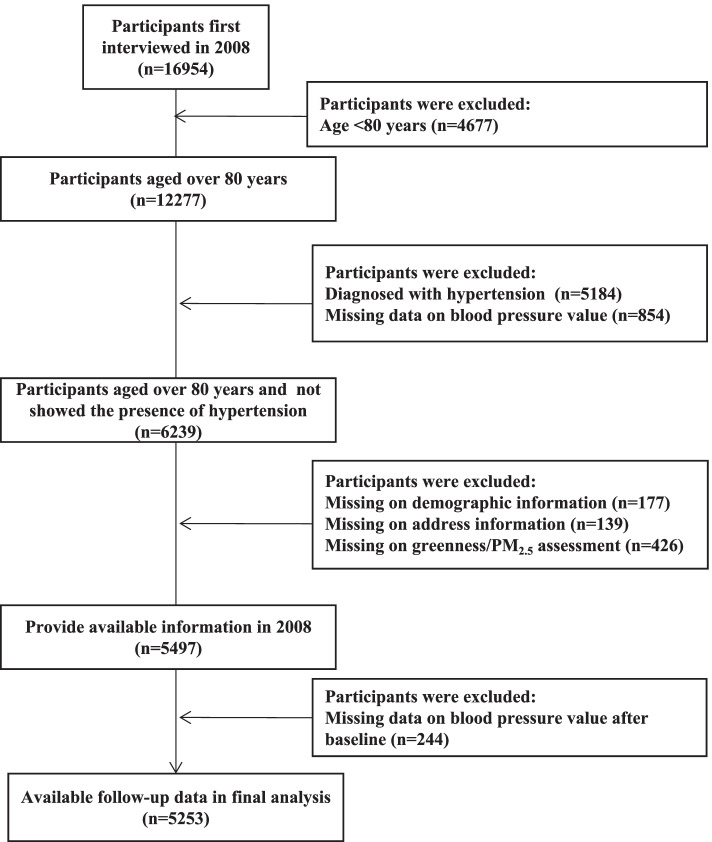
Flow diagram of study including a group of people aged ≥80 years

To protect the participants’ privacy, home address information was removed from this open public database. We selected the fifth (2008) wave as baseline because the information on residential units (at counties/district level) of the residing participants was available from the community investigation questionnaire [22]. At baseline, participants were excluded if they were aged < 80 years, had hypertension (systolic blood pressure [SBP] ≥ 140 mmHg, or diastolic blood pressure [DBP] ≥ 90 mmHg, or normal BP but diagnosed with hypertension at a public hospital), and were missing relevant information (demographic characteristics, BP measurements, and exposure assessment). Finally, the current study consisted of 5253 participants who were first recruited in 2008 and then assessed for events by follow-up surveys implemented in the 2011, 2014, and 2018 waves. A detailed flowchart of the current study is presented in Fig. 1. In our study, the sample population was highly stationary, with only 1.6% of the participants changing their residence (in county/district/rural/urban level) during follow-up; because of their advanced age and the hukou household registration system [23], these individuals were less likely to move to other addresses.

The Research Ethics Committees of Peking University and Duke University granted approval for the Protection of Human Subjects for the CLHLS, including the collection of data used for the present study. The survey respondents provided informed consent prior to their participation.

### Hypertension assessment

As a part of the CLHLS, at baseline and during each interview, the SBP and DBP of the participants were measured at 5-minute rest intervals by a trained physician using a mercurial sphygmomanometer. Through repeated measurements of BP (with 5-minute or longer rest intervals), the average values of SBP and DBP were calculated. Specifically, for bedbound participants, BP measurements were obtained in a recumbent position. Simultaneously, participants were also asked to respond to the following question: “Are you suffering from hypertension that was diagnosed at a public hospital or not?” Thus, in our study, hypertension was defined, according to the guidelines, as SBP ≥ 140 mmHg or DBP ≥ 90 mmHg or normal BP value but self-reported to have been diagnosed with hypertension at a public hospital. A similar study used the same definition of hypertension based on the previously published CLHLS database [24]. In particular, the date of hypertension diagnosis was not clear because the follow-up examinations occurred every 2–3 years for each cohort [25]; we could only determine the results of each follow-up.

### Greenness assessment

The normalized difference vegetation index (NDVI) and enhanced vegetation index (EVI) sourced from the Moderate Resolution Imaging Spectroradiometer (MODIS) Terra were used to estimate greenness. In our study, the values were all based on the cloud-free data of a 16-day recorded period for a 500-m buffer. The two satellite-derived vegetation indices reflected the vegetation of the ground with a range from − 1 to 1. In general, NDVI of 0.1 or below reflected barren areas of rock, sand, or snow; NDVI values between 0.2 and 0.5 generally corresponded to sparse vegetation of row crops, shrubs, and grasslands; and NDVI values between 0.6 and 0.9 usually reflected dense vegetation [26]. A higher value of NDVI/EVI was indicative of denser greenness. Similar to a previous report [27], we considered identifying a window period before survival events that had the greatest impact on hypertension. Given that the participants included in our study were over 80 years old and were likely to drop out owing to death during follow-up, longer exposure estimates were not appropriate, and an adequate sample size might not be warranted. Thus, we examined the relationship between different exposure time windows (baseline, 1-year average, 2-year average, and 3-year average greenness exposure before survival events) and hypertension. Eventually, we decided that exposure to greenness was to be averaged for the 1 year before survival events or censoring, because this time window had the greatest protective impact on hypertension incidence (Table S1). Because of individual privacy protection, exact residence addresses were not made available; thus, the exposure was assessed at a district or county level where older adults resided.

The cloud-free NDVI/EVI values were downloaded from the Google Earth Engine (https://developers.google.cn/earth-engine) and were extracted using ArcGIS 10.6 (ESRI, Redlands, CA, USA) in this study.

### Covariates

Some baseline characteristics were used to control for confounding effects in the study according to prior studies [3, 9]. These included age, gender (female, male), residence (rural/town, urban), pension (yes, no), living arrangements (with family member (s), in a nursing home/alone), education (uneducated, educated), marital status (married/living with a partner, widowed, and single/divorced/separated), current smoking status (yes, no), current drinking status (yes, no), self-reported diabetes (yes, no), ongoing exercising habit (yes, no), and body mass index (BMI) (< 18.5, 18.5–23.9, > 23.9). The categories of the BMI were according to the Chinese recommendation for BMI cutoffs. In particular, we used the Qinling Mountain-Huai River line to divide residential units into two regions, northern China (Beijing, Tianjin, Hebei, Henan, Shandong, Shanxi, Shaanxi, Heilongjiang, Jilin, and Liaoning Provinces) and southern China (Fujian, Jiangsu, Hunan, Hubei, Zhejiang, Guangdong, Hainan, Guangxi, Anhui, Jiangxi, Shanghai, Chongqing, and Sichuan Provinces).

In accordance with prior studies, it was also important to control for the effect of air pollution. Hence, we selected the annual average PM_2.5_ concentrations at baseline as confounding variables in the analysis. Data on the baseline PM_2.5_ concentration in 652 units where the participants resided were collected from an open public database built by the Atmospheric Composition Analysis Group from the University of Washington [28]. Researchers used satellite combining Aerosol Optical Depth (AOD) retrievals from multiple satellite products (MISR, MODIS Dark Target, MODIS and SeaWiFS Deep Blue, and MODIS MAIAC) to report ground-level PM_2.5_ concentrations with a 1.1 × 1.1 km resolution.

In addition, the leisure activity of each individual was investigated in the CLHLS project, which included the following: housework, personal outdoor activities (doing fieldwork/practicing labor activity), gardening, reading newspapers/books, raising domestic animals, playing cards and/or mah-jongg, watching television and/or listening to the radio, and organized activities. Each response was scored on a scale of 1 (never), 2 (not every month but sometimes), 3 (not every week but at least once a month), 4 (not every day but at least once a week), or 5 (almost every day). We selected two indicators related to outdoor activities (i.e., personal outdoor activities and gardening) as important variables in the analysis. Given the size of the sample, personal outdoor activities and gardening were categorized as binary variables and reported as 1 (never/sometimes) and 2 (at least once a month or more) [29].

### Statistical analysis

First, descriptive statistical analysis was performed. Continuous variables with normal distribution were reported as mean ± standard deviation (SD). The median and interquartile range (IQR) were presented for variables with a non-normal distribution. Categorical variables were reported as numbers and percentages. In the survival analysis, the duration of follow-up was from the interview date participants were enrolled in the project to the interview date of incident hypertension or end of follow-up or administrative censoring date (identified for those lost to follow-up as the middle date between the last survey when the participant was interviewed and the subsequent survey), whichever occurred first. Person-years were calculated using a precise method (days/365) [23].

We initially explored the exposure-response relationships between greenness exposure and hypertension incidence by using Cox proportional hazards regression models of penalized splines with different degrees of freedom after adjusting for covariates [30]. In this study, based on the minimum Akaike information criterion (AIC) value, we set the degrees of freedom as 4. As a robustness check, to verify the linear or nonlinear effects of residential greenness exposure on hypertension incidence, we performed sensitivity analyses by excluding participants who had changed address or dropped out owing to death during the follow-up period. We also performed sensitivity analyses using NDVI and EVI values with 250-m and 1000-m buffer radii. In addition, we further performed sensitivity analyses by adjusting for PM_2.5_ concentrations contemporaneously with greenness exposure.

Using penalized splines with 4 dfs, the exposure-response curves showed that the association between greenness and hypertension was not linear. Herein, we used a Cox proportional hazards model included a random effect term to assess the linear relationship between each 0.1-unit increase in greenness exposure and hypertension incidence. Random-effects Cox proportional hazards models were used because of the set clustering of the study designs. In this analysis, the residential units of the participants were included as a random effect [31]. The models were fitted after stratifying the data according to the greenness exposure change points (i.e., 0.30 for NDVI; 0.21 for EVI), which were critically extracted from the shapes of the exposure-response curves [30]. The crude model only included NDVI/EVI (Model 1). Model 2 was developed by further adjusting for age, gender, residence, geographical regions, pension, living arrangements, education, marital status, smoking status at present, drinking status at present, ongoing exercising habits, self-reported diabetes, PM_2.5_ concentration, gardening, personal outdoor activities, and BMI.

In accordance with prior studies [32–34], we evaluated potential modifiers of an association between greenness and hypertension such as gender, residence, geographical regions, smoking, drinking, ongoing exercising habit, gardening, and personal outdoor activities in each stratum divided by change points. The interaction effects of NDVI/EVI and subgroup variables on hypertension incidence were tested by performing a cross-product analysis.

Effect estimates are shown as hazard ratios (HRs) with the corresponding 95% confidence intervals (CIs). The map of China was derived from the National Geomatics Center of China (http://www.ngcc.cn/ngcc/), and the statistics map was generated using ArcGIS Geospatial Analyst module v10.6 (ESRI, Redlands, CA, USA). A two-tailed *p*-value less than 0.05 was considered statistically significant. All analyses were performed with R (version 4.0.5; R Development Core Team).

## Results

Demographic characteristics of the participants are presented in Table 1. Among the 5253 participants, 1048 new cases of hypertension were observed, including 14,454.64 person-years of follow-up, with an incidence rate of 7.25 (95% CI: 6.83–7.67) per 100 person-years. The mean age of the participants was 93.7 years (SD = 7.16), with a range of 81–116 years. More than half of the participants (60.9%) were women. Only 28.4% of the participants were educated, and 80.9% were living in town/rural areas; most were widowed (81.7%) and living with family member (s) (83.4%). The majority of the participants did not receive a pension (Table 1).

**Table 1:** Sociodemographic characteristics of study population at baseline (*N* = 5253)

Variables	Overall	n(%)/mean ± SD
Age		93.7 ± 7.16
Gender		
	Female	3201 (60.9)
	Male	2052 (39.1)
Education attainment		
	Uneducated	3763 (71.6)
	Educated	1490 (28.4)
Pension		
	No	4525 (86.1)
	Yes	728 (13.9)
Residence		
	Rural/Town	4251 (80.9)
	Urban	1002 (19.1)
Living arrangement		
	Nursing home/alone	871 (16.6)
	Living with family member (s)	4382 (83.4)
Region		
	Northern China	1236 (23.5)
	Southern China	4017 (76.5)
Marital status		
	Single/divorced/separated	57 (1.1)
	Widow	4294 (81.7)
	Married/living together	902 (17.2)
Smoking status at present		
	No	4517 (86.0)
	Yes	736 (14.0)
Drinking status at present		
	No	4409 (83.9)
	Yes	844 (16.1)
Ongoing exercising habit		
	No	4089 (77.8)
	Yes	1164 (22.2)
Self-reported diabetes		
	No	5184 (98.7)
	Yes	69 (1.3)
BMI		
	< 18.5	2236 (42.6)
	18.5–23.9	2608 (49.6)
	> 23.9	409 (7.8)
Leisure activity		
Personal outdoor activities		
	Never/sometimes	2775 (52.8)
	At least once a month and more	2478 (47.2)
Gardening		
	Never/sometimes	4918 (93.6)
	At least once a month and more	335 (6.4)

**Fig. 2:**
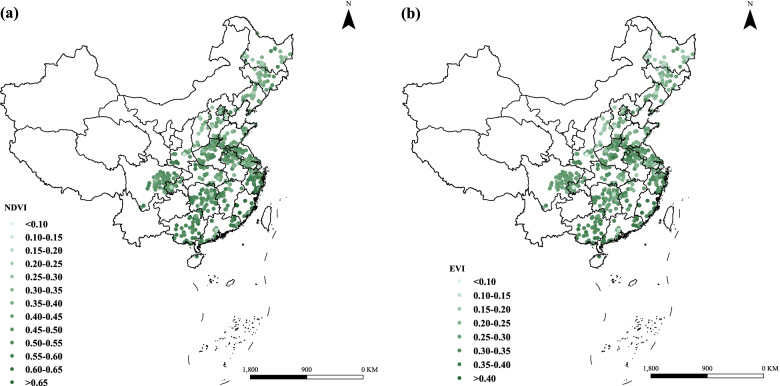
Distributions of greenness indices on 652 units at baseline for 5253 participants. a NDVI and b EVI

The spatial distribution of NDVI/EVI values at baseline in 652 units among 5253 oldest-old adults in China is presented in Fig. 2. As seen in Fig. 2, we found that the gradient of greenness seemed to follow the north-south axis. We also presented the spatial distribution of the NDVI/EVI values at baseline across China (Fig. S1). We could see that the IQRs of NDVI and EVI were 0.15 and 0.09 with medians of 0.48 and 0.30, respectively. The participants were exposed to a PM_2.5_ median concentration of 51.81 μg/m^3^ (IQR = 25.24 μg/m^3^) (Table 2).

**Table 2:** Descriptive statistics analysis for vegetation indices and air pollutant

Variables	Mean	SD	Min	Quantiles			Max	IQR
				P_25_	P_50_	P_75_		
EVI	0.29	0.07	0.05	0.25	0.30	0.34	0.44	0.09
NDVI	0.46	0.11	0.09	0.39	0.48	0.54	0.69	0.15
PM_2.5_ concentration (μg/m^3^)	53.93	16.00	9.44	42.78	51.81	68.02	95.60	25.24

**Fig. 3:**
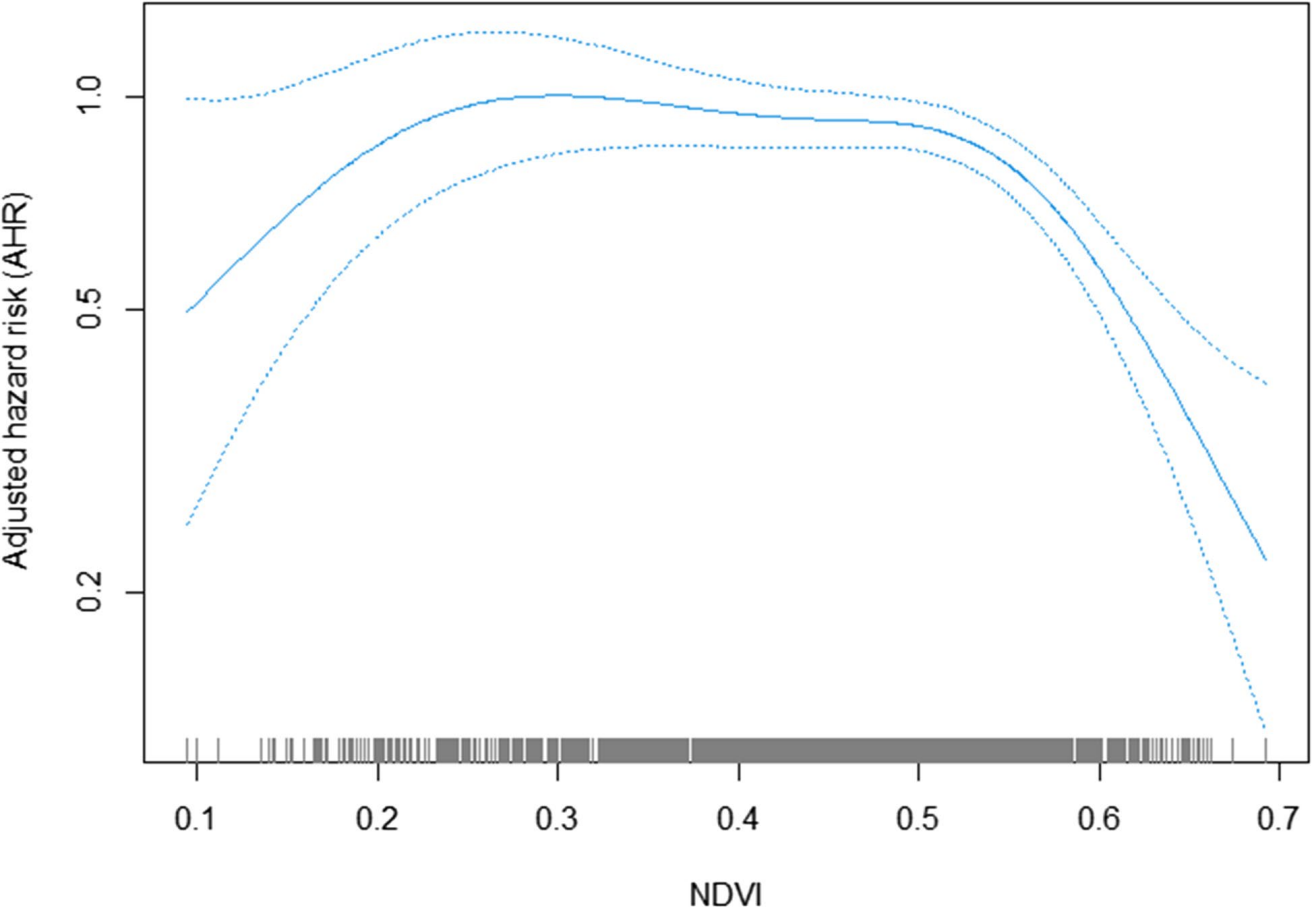
Association of NDVI and hypertension incidence risk in Cox models with penalized splines (0.30: reference). Adjusted for age, living arrangement, PM_2.5_ concentration, regions, residence, gender, smoking, drinking, exercising, pension, marital status, education attainment, self-reported diabetes, personal outdoor activities, gardening, and BMI

**Fig. 4:**
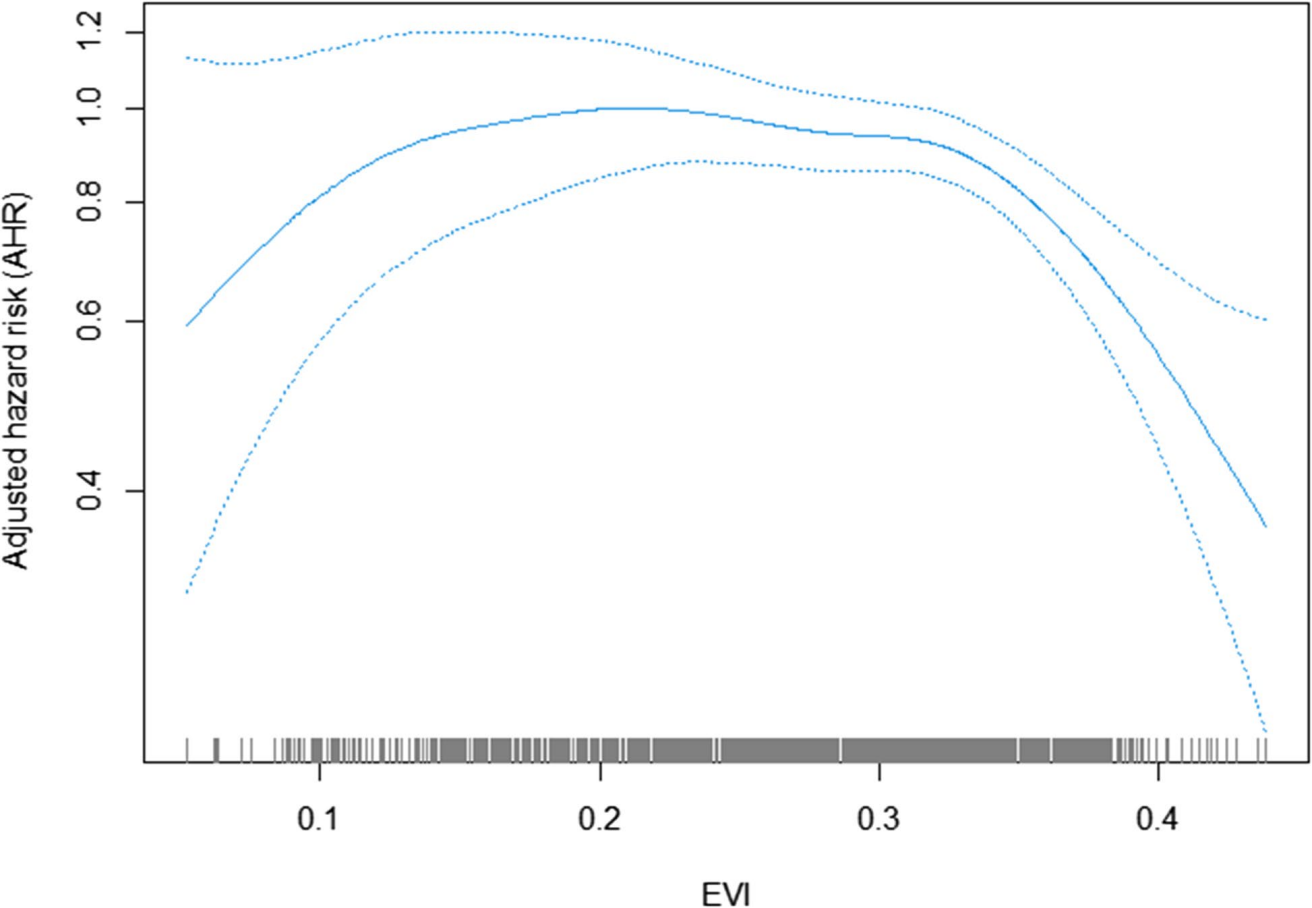
Association of EVI and hypertension incidence risk in Cox models with penalized splines (0.21: reference). Adjusted for age, living arrangement, PM_2.5_ concentration, regions, residence, gender, smoking, drinking, exercising, pension, marital status, education attainment, self-reported diabetes, personal outdoor activities, gardening, and BMI

Figures 3–4 show the exposure-response association between NDVI/EVI and hypertension risk. The shape of the curves indicated that there were nonlinear links between greenness exposure and hypertension risk (*P*-value for nonlinearity test for NDVI was < 0.001; *P*-value for nonlinearity test for EVI was 0.001) and was presented as an inverse “U-shape”. The nonlinear association was still significant after excluding participants who changed addresses and dropped out owing to death during follow-up and using NDVI/EVI values with 250 and 1000-m buffer radii as well as adjusted for PM_2.5_ concentrations contemporaneously with greenness exposure (Figs. S2–S5). According to the shape of the exposure-response curve, the change point of NDVI for hypertension was 0.30. The hypertension risk increased as far as 0.30; the risk declined until the end, albeit many parts of the confidence intervals did overlap the null. The change point in the EVI for hypertension was 0.21; namely, the hypertension risk first increased until 0.21 and then decreased until the end. However, we could see many parts of the confidence intervals did overlap the null also. After that, we used the Cox proportional hazards model included a random effect term to examine the linear relationship between greenness exposure and hypertension risk. We stratified participants into those living in areas with low (i.e., NDVI/EVI value ≤ change point) and high levels (i.e., NDVI/EVI value > change point) of greenness based on the change points. Table 3 summarizes the results for the linear association between greenness and hypertension. After adjusting for related covariates, a strong protective effect of greenness exposure on hypertension existed only in those with high-level greenness exposure (HR = 0.60; 95% CI: 0.53–0.67 for NDVI; HR = 0.46, 95% CI: 0.37–0.57 for EVI). However, no significant associations were detected when the greenness was lower than the change point (HR = 0.77; 95% CI: 0.38–1.55 for NDVI; HR = 0.73; 95% CI: 0.33–1.63 for EVI).

**Table 3:** Association between per 0.1-unit increase in vegetation index and hypertension prevalence

Index	NDVI				EVI			
	Low-level exposure (*n =* 594)	AIC	High-level exposure (*n =* 4659)	AIC	Low-level exposure (*n =* 799)	AIC	High-level exposure (*n =* 4454)	AIC
Model 1	1.30 (0.78–2.18)	913.2	**0.77** **(0.69–0.86)**	13,577.4	1.16 (0.59–2.26)	1218.0	**0.58** **(0.48–0.70)**	13,160.1
Model 2	0.77 (0.38–1.55)	911.8	**0.60** **(0.53–0.70)**	13,509.5	0.73 (0.33–1.63)	1217.1	**0.46** **(0.37–0.57)**	13,112.9

*Note:*

*Values indicated in bold are statistically significant*

Model 1: Crude model only included NDVI/EVI

Model 2: Crude model + further adjusted for age, living arrangement, PM_2.5_ concentration, regions, residence, gender, smoking, drinking, exercising, pension, marital status, education attainment, self-reported diabetes, personal outdoor activities, gardening, and BMI

In the subgroup analysis, we first examined the interaction effect between modifiers and NDVI/EVI on hypertension at each level of the exposure group. No significant associations between greenness exposure and hypertension risk were found in any subgroups (Tables 4–5).

**Table 4:** Association between per 0.1-unit increase in NDVI and hypertension incidence, stratified by subgroup factors

Variables	Overall	High-level exposure (*n =* 4659)		Low-level exposure (*n =* 594)	
		HR(95% CI)	*P* for interaction	HR(95% CI)	*P* for interaction
Gender					
	Female	0.62 (0.53–0.73)	0.740	0.62 (0.23–1.65)	0.780
	Male	0.71 (0.60–0.85)		0.75 (0.22–2.48)	
Residence					
	Rural/Town	0.58 (0.51–0.67)	0.760	7.17 (0.21–240.80)	0.260
	Urban	0.67 (0.43–1.05)		0.56 (0.26–1.20)	
Region					
	Northern China	0.78 (0.56–1.09)	0.120	0.44 (0.14–1.34)	0.210
	Southern China	0.52 (0.45–0.61)		0.71 (0.22–2.28)	
Smoking status at present					
	No	0.61 (0.53–0.70)	0.160	0.73 (0.34–1.56)	0.610
	Yes	0.62 (0.45–0.84)		0.69 (0.03–14.75)	
Drinking status at present					
	No	0.64 (0.56–0.74)	0.110	0.51 (0.23–1.15)	0.073
	Yes	0.60 (0.46–0.78)		3.07 (0.33–28.25)	
Ongoing exercising habit					
	No	0.62 (0.54–0.72)	0.930	1.16 (0.43–3.13)	0.100
	Yes	0.68 (0.56–0.83)		0.60 (0.23–1.58)	
Leisure activity					
Personal outdoor activities					
	Never/sometimes	0.64 (0.53–0.77)	0.420	3.11 (0.45–22.10)	0.210
	At least once a month and more	0.67 (0.56–0.77)		0.51 (0.22–1.18)	
Gardening					
	Never/sometimes	0.60 (0.53–0.69)	0.790	1.00 (0.46–2.15)	0.830
	At least once a month and more	0.64 (0.38–1.09)		0.14 (0.01–1.95)	

*Note:*

The associations were adjusted by age, living arrangement, PM_2.5_ concentration, regions, residence, gender, smoking, drinking, exercising, pension, marital status, education attainment, self-reported diabetes, personal outdoor activities, gardening, and BMI (unless stratified by the respective factor)

**Table 5:** Association between per 0.1-unit increase in EVI and hypertension incidence, stratified by subgroup factors

Variables	Overall	High-level exposure (*n =* 4454)		Low-level exposure (*n =* 799)	
		HR(95% CI)	*P* for interaction	HR(95% CI)	*P* for interaction
Gender					
	Female	0.53 (0.41–0.68)	0.480	0.95 (0.32–2.87)	0.240
	Male	0.55 (0.42–0.74)		0.64 (0.18–2.27)	
Residence					
	Rural/Town	0.43 (0.35–0.54)	0.400	5.07 (0.20–13.01)	0.110
	Urban	0.71 (0.31–1.60)		0.53 (0.23–1.26)	
Region					
	Northern China	0.67 (0.39–1.17)	0.180	0.45 (0.15–1.33)	0.074
	Southern China	0.39 (0.31–0.50)		1.05 (0.23–4.87)	
Smoking status at present					
	No	0.48 (0.38–0.60)	0.410	0.77 (0.34–1.75)	0.890
	Yes	0.52 (0.32–0.84)		1.65 (0.10–28.20)	
Drinking status at present					
	No	0.53 (0.42–0.66)	0.130	0.55 (0.23–1.34)	0.095
	Yes	0.48 (0.32–0.71)		1.14 (0.03–37.92)	
Ongoing exercising habit					
	No	0.50 (0.39–0.62)	0.580	1.04 (0.36–3.02)	0.400
	Yes	0.59 (0.42–0.84)		0.69 (0.23–2.07)	
Leisure activity					
Personal outdoor activities					
	Never/sometimes	0.47 (0.35–0.64)	0.990	3.85 (0.53–27.81)	0.310
	At least once a month and more	0.58 (0.44–0.75)		0.79 (0.34–1.79)	
Gardening					
	Never/sometimes	0.44 (0.35–0.55)	0.310	1.18 (0.49–2.85)	0.520
	At least once a month and more	0.93 (0.37–2.34)		0.20 (0.03–1.40)	

*Note:*

The associations were adjusted by age, living arrangement, PM_2.5_ concentration, regions, residence, gender, smoking, drinking, exercising, pension, marital status, education attainment, self-reported diabetes, personal outdoor activities, gardening, and BMI (unless stratified by the respective factor)

## Discussion

To our knowledge, this is the first prospective cohort study to examine the association between exposure to greenness and hypertension in the Chinese oldest-old population. The findings from our study showed that the dose-response curve for greenness exposure with hypertension risk was not linear, and 1 change point was detected. We found that greenness exposure higher than the change point (i.e., high-level exposure) had a greater protective effect on the risk of hypertension incidence than low-level exposure.

One of the highlights of our study was that we observed an inverted “U-shaped” dose-response relationship between greenness exposure and hypertension. The shape of the exposure-response curve was similar to that in several published surveys that implemented nonlinear approaches to detect associations between greenness and physical function, overweight, birth outcomes, antidepressant prescription rates, self-reported mental status, and mortality of the elderly [35–40]. Among them, four investigations showed green-space correlations that had the unexpected positive association before change point [35, 36, 38, 39]. Another two showed that greenness was negatively associated with improved birth outcomes or low risk of mortality of the elderly when participants were exposed to high levels of greenness [37, 40]. Regarding hypertension, most of the studies suggested linear and protective effects of greenness on hypertension, but some of them also provided possible evidence for nonlinear links. For instance, an earlier study indicated that moderate levels of greenness appeared to increase BP more than low levels [41]. Yang et al. reported that the third quantile of greenness exposure was more related to higher SBP value than the first quantile [10]. Jendrossek et al. proposed that hypertension risk in the medium level of greenness exposure was higher than in the low-level exposure [42]. The results of two recent cross-sectional studies conducted in Lithuania and Madrid showed that the probability of hypertension did not present a monotone decreasing trend with increased exposure to higher proportions of green space in the residential address [43, 44].

In the current study, we found that the effect on hypertension risk was not significant at low-level exposure; in turn, the hypertension risk declined at high-level exposure. Taking NDVI as an example, the mean value in low-level exposure was 0.22, which generally corresponded to sparse vegetation or mixed land use or built areas. It may not be attractive for residents and negates the health benefits of green space [35, 45]. These results provide a novel hypothesis that greenness exposure may need to reach a certain level before strong health effects are observed [46]. Nonetheless, owing to the limited evidence of the nonlinear correlation between greenness and hypertension, in-depth studies should be conducted to verify whether this association is reliable and accurate.

Our findings are supported by those of prior studies that revealed the protective effects of greenness exposure on the incidence of hypertension [41, 47, 48]. However, few studies have included oldest-old adults. In an earlier community-based cross-sectional study of participants aged 18–91 years, approximately 45% of the risk of hypertension among adults > 65 years was reduced by greenness exposure [17]. One observational survey of five locations in central China found that greenness exposure was associated with an approximately 5% lower risk of hypertension in the middle-aged and older populations (mean age: 55.58 years) [3]. A study of 249,405 Medicare beneficiaries from Florida revealed a 13% decrease in risk of hypertension attributed to greenness among adults aged 65–111 years [49]. In addition, a recent study from 33 communities in China indicated that greenness exposure seemed to be a risk factor for hypertension in adults aged > 65 years (accounting for 9% of their sample), but no statistical significance was detected [10]. Because the total number of oldest-old individuals (aged ≥ 80 years) was very limited, our data were collected by national investigations to fill existing study gaps in past literature, with implications for illustrating the benefit of greenness on hypertension and a deeper understanding of longevity. The significant association between greenness exposure and hypertension incidence indicates that improvement in residential green space may be helpful to supplement existing guidelines for the management of geriatric hypertension [50]. At high levels of greenness, we found that each 0.1-unit increment in NDVI and EVI was associated with 40 and 54% lower odds of hypertension, respectively. The results of our study showed a greater protective effect than that observed in previous studies on older adults [10, 12, 14, 17]. Thus, it is of significant public health interest to expand extensive green spaces and offer access to green spaces in nursing homes, geriatric hospitals, and residential areas that are designated for the oldest-old (e.g., longevity villages), which can reduce age-specific hypertension occurrence and increase life expectancy [51].

Previous studies have suggested the following factors that may contribute to the underlying mechanisms for the influence of greenness on hypertension: 1) Exposure to greenness can alleviate emotional symptoms and the pressure faced by an individual [52, 53]; 2) Green spaces offer accessible opportunities for physical activity and social interaction for individuals [51, 54]; and 3) Noise, air pollutants, heat, and dust exposure can be offset to some extent by a large green space [55]. Weight control, physical activity, and reducing mental pressure are important prevention strategies included in current recommendations based on guidelines for the management of geriatric hypertension. The current study also emphasizes the need to update recommendations for the prevention of hypertension owing to our advancing understanding of the environmental factors for hypertension incidence.

This study had several limitations. First, the precise residential addresses of the participants were not available because of the privacy protection of the CLHLS. Under this condition, we used county/district level exposure information to reflect the exposure of an individual. Although the same method was applied in prior studies [56, 57], measurement errors existed to some extent. However, we used satellite data with a high-resolution and national-level representative sample to enhance the reliability and generalizability of our findings. In addition, previous residential units-based surveys to examine the effects of greenness exposure on health outcomes presented positive results, which support the scientific merit of our results [58–61]. Second, limited by the CLHLS project database, data describing the greenness vegetation structure, vegetation quality, and specific vegetation types were not available. Further study should be conducted to examine the influence of the dynamic seasonality of greenness on health outcomes. Third, the baseline CLHLS project data lacked information on the quantitative measurement of physical activity (intensity and frequency); also, data on the exact onset of hypertension were unavailable. Therefore, the generalizability of our results is also limited to some extent.

The strengths of our study in the estimation of the effect of greenness on hypertension among adults of 80 years of age or more include large sample size and prospective cohort design. The study also underlined the importance of continued expansion of residential green spaces. Finally, greenness was independent of conventional hypertension risk factors. We expect that a comprehensive assessment of the environmental factors associated with hypertension risk is needed for older adults. We also recommend that the existing guidelines for hypertension prevention should be updated, and physicians should be aware of these environmental factors and inform their patients of the potential risk management measures they can adopt.

## Conclusion

A representative sample of the oldest-old population was analyzed in the current study to investigate the protective effects of greenness exposure on hypertension incidence, and it was found that this exposure-response association was not linear but reflected an inverse “U-shaped” curve. Our study emphasizes the importance of expanding green spaces to reduce hypertension risk; however, further studies are needed to confirm the generalizability of our findings.

## Supplementary Information

**Additional file 1.**

## Data Availability

The CLHLS is an open-publish database which can be downloaded at website http://cnsda.ruc.edu.cn/index.php?r=projects/view&id=52572397.

## Abbreviations

CLHLS: Chinese Longitudinal Healthy Longevity Survey
BP: Blood pressure
CVD: Cardiovascular disease
SBP: Systolic blood pressure
DBP: Diastolic blood pressure
NDVI: Normalized difference vegetation index
EVI: Enhanced vegetation index
MODIS: Moderate Resolution Imaging Spectroradiometer
AOD: Aerosol Optical Depth
HRs: Hazard ratios
CIs: Confidence intervals
SD: Standard deviation
IQR: Interquartile range
BMI: Body mass index
PM: Fine particulate matter
