# Supplementary material for: The effects of greenness exposure on hypertension incidence among Chinese oldest-old: a prospective cohort study
Source: Environ Health. 2022 Jul 11;21:66. doi: 10.1186/s12940-022-00876-6 (PMC9277785; doi:10.1186/s12940-022-00876-6)
Supplement: Supplementary file 1 — Additional file 1. [file 12940_2022_876_MOESM1_ESM.pdf]

Supplement materials

The effects of greenness exposure on hypertension incidence among Chinese oldest-old: a prospective cohort study

Contents

Figure S1 The spatial distribution of vegetation index at baseline across China

Table S1 Window periods analysis for the association between per 0.1-unit increment in vegetation index and hypertension

Figure S2 Sensitivity analysis for the association between EVI_{500m} and hypertension incidence risk in Cox models with penalized splines

Figure S3 Sensitivity analysis for the association between $NDVI_{500m}$ and hypertension incidence risk in Cox models with penalized splines

Figure S4 Association of $NDVI_{250m}$ and EVI_{250m} and hypertension incidence risk in Cox models with penalized splines

Figure S5 Association of $NDVI_{1000m}$ and EVI_{1000m} and hypertension incidence risk in Cox models with penalized splines

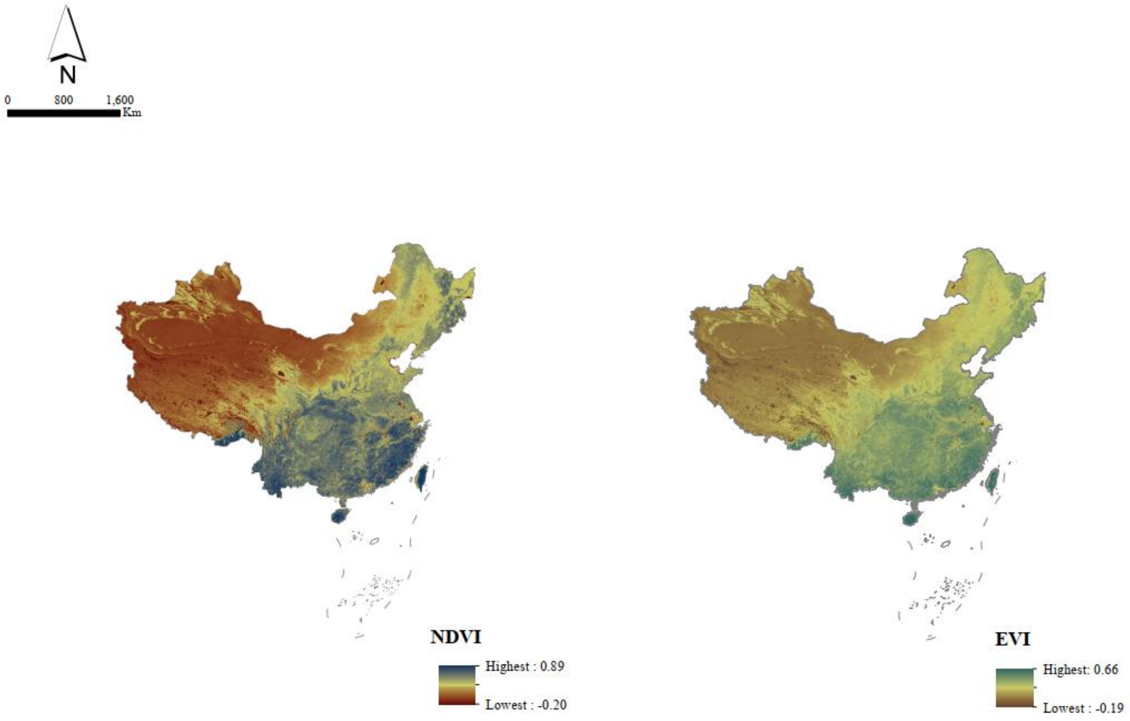

The spatial distribution of NDVI at baseline across China

The spatial distribution of EVI at baseline across China

Fig. S1 The spatial distribution of vegetation index at baseline across China

Table S1 Window periods analysis for the association between per 0.1-unit increment in vegetation index and hypertension [a]

	NDVI	EVI
Average 3-year exposure	1.01(0.92-1.12)	1.04(0.90-1.21)
Average 2-year exposure	0.99(0.90-1.09)	0.99(0.86-1.15)
Average 1-year exposure	**0.76 (0.69-0.84)**	**0.71 (0.61-0.82)**
Baseline exposure	1.07(0.97-1.19)	1.14(0.97-1.33)

Note:

Values indicated in bold were statistically significant.

[a] After adjusted for age, living arrangement, $PM_{2.5}$, regions, residence, gender, smoking, drinking, exercising, pension, marital status, education attainment, self-reported diabetes, personal outdoor activities, gardening and BMI.

(A)

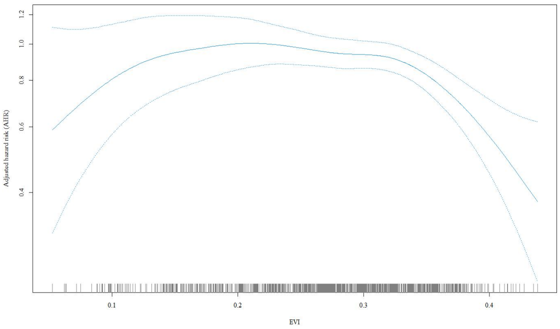

(B)

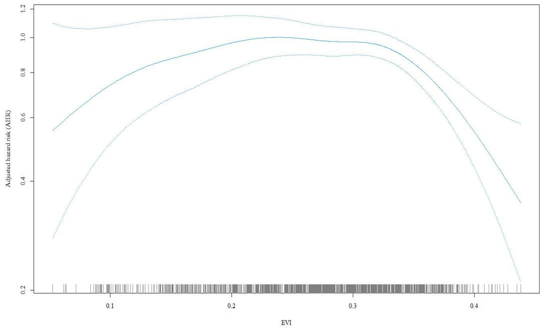

(C)

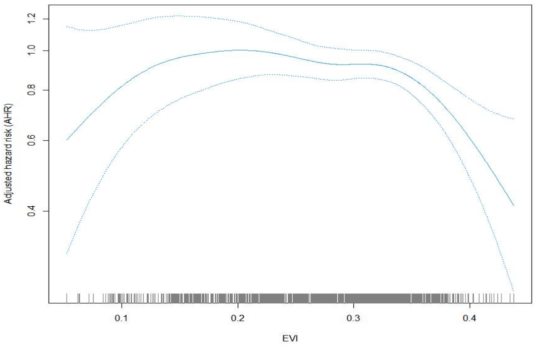

Fig. S2 Sensitivity analysis for the association between EVI_{500m} and hypertension incidence risk in Cox models with penalized splines (change point as reference) [a]. **A** The analysis was conducted after excluding participants who died during follow-up (*P*-value for nonlinearity test was <0.001; with its degrees of freedom set to 4), **B** The analysis was conducted after excluding participants who changed addresses (*P*-value for nonlinearity test was 0.001; with its degrees of freedom set to 4). **C** The analysis was conducted adjusted for $PM_{2.5}$ concentrations contemporaneously with greenness exposure. (*P*-value for nonlinearity test was 0.008; with its degrees of freedom set to 4). [a] Adjusted for age, living arrangement, $PM_{2.5}$, regions, residence, gender, smoking, drinking, exercising, pension, marital status, education attainment, self-reported diabetes, personal outdoor activities, gardening and BMI.

(A)

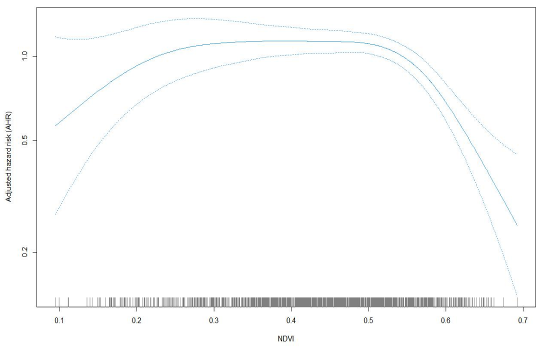

(B)

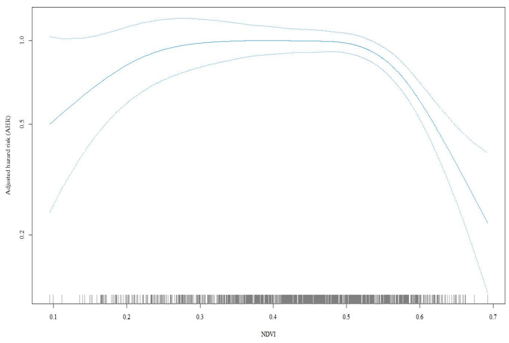

(C)

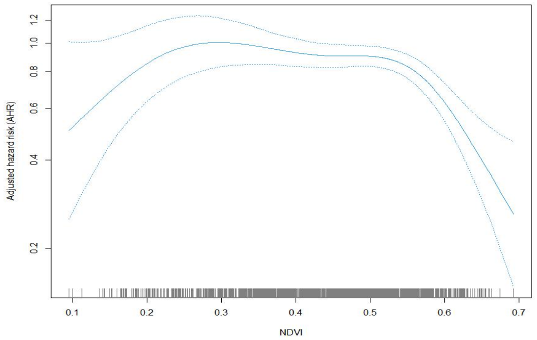

Fig. S3 Sensitivity analysis for the association between $NDVI_{500m}$ and hypertension incidence risk in Cox models with penalized splines (change point as reference) [a]. **A** The analysis was conducted after excluding participants who died during follow-up (*P*-value for nonlinearity test was 0.002; with its degrees of freedom set to 4), **B** The analysis was conducted after excluding participants who changed addresses (*P*-value for nonlinearity test was <0.001; with its degrees of freedom set to 4). **C** The analysis was conducted adjusted for $PM_{2.5}$ concentrations contemporaneously with greenness exposure. (*P*-value for nonlinearity test was <0.001; with its degrees of freedom set to 4). [a] Adjusted for age, living arrangement, $PM_{2.5}$, regions, residence, gender, smoking, drinking, exercising, pension, marital status, education attainment, self-reported diabetes, personal outdoor activities, gardening and BMI.

(A) (B)

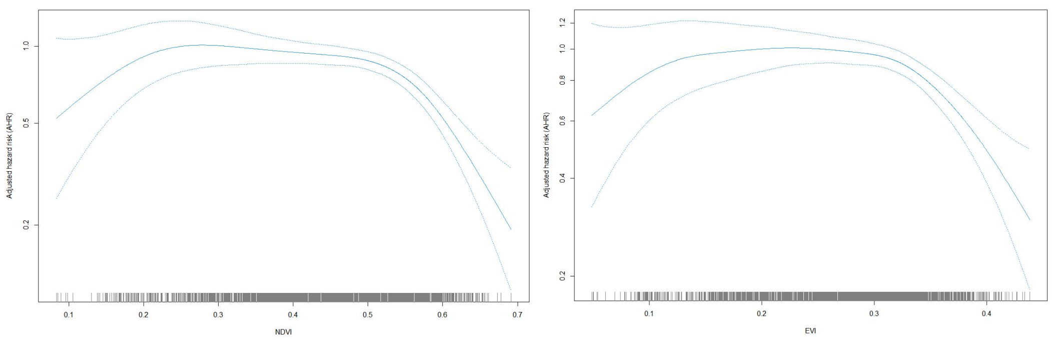

Fig. S4 Association of $NDVI_{250m}$ and EVI_{250m} and hypertension incidence risk in Cox models with penalized splines (change point as reference) [a]. **A** $NDVI_{250m}$ (*P*-value for nonlinearity test was <0.001; with its degrees of freedom set to 4), **B** EVI_{250m} (*P*-value for nonlinearity test was <0.001; with its degrees of freedom set to 4). [a] Adjusted for age, living arrangement, $PM_{2.5}$, regions, residence, gender, smoking, drinking, exercising, pension, marital status, education attainment, self-reported diabetes, personal outdoor activities, gardening and BMI.

(A) (B)

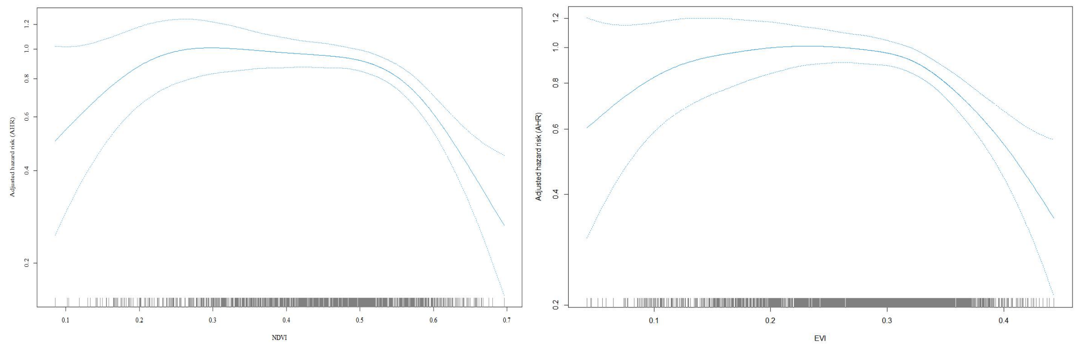

Fig. S5 Association of $NDVI_{1000m}$ and EVI_{1000m} and hypertension incidence risk in Cox models with penalized splines (change point as reference) [a]. **A** $NDVI_{1000m}$ (*P*-value for nonlinearity test was <0.001; with its degrees of freedom set to 4), **B** EVI_{1000m} (*P*-value for nonlinearity test was <0.001; with its degrees of freedom set to 4). [a] Adjusted for age, living arrangement, $PM_{2.5}$, regions, residence, gender, smoking, drinking, exercising, pension, marital status, education attainment, self-reported diabetes, personal outdoor activities, gardening and BMI.
